# Lck activation: puzzling the pieces together

**DOI:** 10.18632/oncotarget.22309

**Published:** 2017-11-07

**Authors:** Luca Simeoni

**Affiliations:** Luca Simeoni: Otto-von-Guericke University, Institute of Molecular and Clinical Immunology, Magdeburg, Germany

**Keywords:** Lck, T-cell activation, TCR signaling, conformational dynamics, tyrosine phosphorylation

T cells are central players in the immune defense, but if deregulated they may also mediate the development of pathological conditions and diseases. Therefore, how T-cell functions are regulated is the focus of intense research since many years. Important steps forward in our understanding of the molecular mechanisms underlying T-cell biology have been made recently. These advances have allowed the development of new strategies to reprogram or to modulate T-cell functions for the treatment of patients suffering from cancer, chronic inflammatory diseases, and autoimmunity.

The Src-family kinase Lck is an essential regulator of T-cell functions (e.g. development, activation, and homeostasis), which is involved in the initiation of TCR signaling. Upon TCR engagement, Lck phosphorylates tyrosine residues within the immunoreceptor tyrosine-based activation motifs (ITAMs) of the TCR-associated CD3 chains. Phosphorylated ITAMs act as docking site for the tandem SH2 domains of the tyrosine kinase Zap-70, which is in turn activated by Lck. Signaling is further propagated upon phosphorylation of the transmembrane adaptor protein LAT by Zap-70. LAT links the TCR to the activation of intracellular signaling cascades leading to the transcription of target genes required for the execution of the appropriate T-cell response. As all members of the Src-family, Lck activity is regulated by two major phosphorylation sites Tyr^394^ and Tyr^505^. Trans- auto-phosphorylation of Tyr^394^ in the kinase domain promotes an open/active conformation, whereas phosphorylation of Tyr^505^, located at the C-terminus, by the tyrosine kinase Csk results in a close/inactive enzyme.

Recently, the regulation of Lck activity has been at the center of intense debates whether TCR triggering induces *de novo* phosphorylation/activation of Tyr^394^ and opening of Lck or not [1-5]. Using both biophysical and biochemical approaches, two studies came to the conclusion that Lck does not open (i.e. is not activated) [1] and that the enzymatic activity of Lck does not change upon TCR stimulation [2]. Based upon these data, the so called “standby” model was proposed, which postulates that TCR stimulation does not induce *de novo* activation/opening of Lck and that ITAM-phosphorylation is likely initiated by a pool of active Lck, which is present in resting T cells [2].

Conversely to the view described above, using an Lck biosensor and fluorescence lifetime imaging (FLIM)/Förster resonance energy transfer (FRET) analyses, our group has found that T-cell stimulation results in a conformational opening of about 20% of Lck molecules at the engaged TCR [3]. The microscopic observations were further corroborated by biochemical data (*in vitro* kinase assays) showing that TCR stimulation increased Lck activity. Therefore, we concluded that *de novo* activation and opening of Lck are required for the initiation of TCR signaling (“*de novo* activation” model).

**Figure 1:**
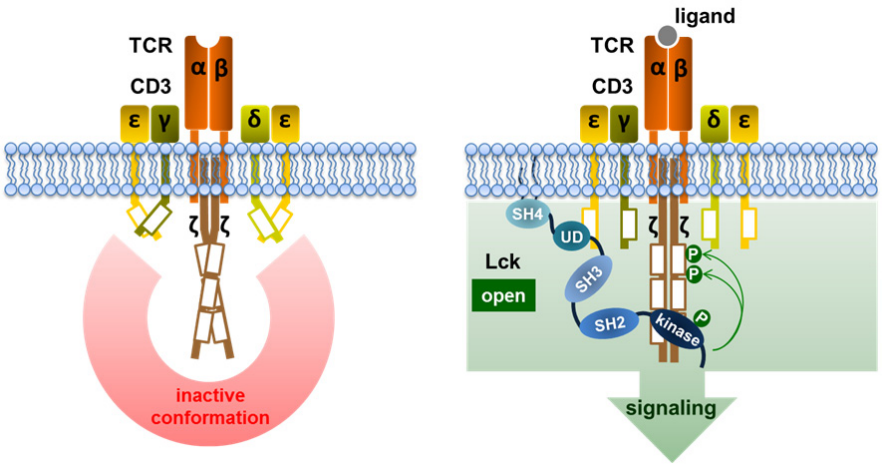
Model for the initiation of TCR signaling In resting conditions, the TCR is in an inactive conformation (left panel). Upon ligand binding (right panel), the following events are induced: (1) TCR is stabilized in an active conformation, (2) Lck is recruited to the TCR/CD3 complex, (3) binding of Lck to CD3ε results in its opening and activation by phosphorylation on Y394, (4) phosphorylation of the ITAMs by Lck initiates TCR signaling. The α and β chains of the TCR as well as CD3ε, CD3δ, CD3γ, and CD3ζ are depicted. A schematic representation of Lck structure including the N-terminal SH4 domain containing lipid modification motifs that are required for membrane association, the unique domain (UD), SH3, SH2, and kinase domains is represented (right panel). Green circles represent tyrosine phosphorylations, whereas the white boxes represent the ITAMs.

To elucidate in more detail how Lck is regulated, we decided to conduct a follow-up study. To this end, the Lck biosensor was optimized to be better suited for FLIM/FRET analyses and additionally an immunofluorescence microscopy protocol was established that allows accurate analyses of the subcellular localization of Lck. Using these sophisticated tools, we most recently demonstrated that Lck undergoes opening and that phosphorylation on Tyr^394^ occurs at the plasma membrane upon TCR stimulation [5], thus confirming our previous data [3]. In our study we made two additional important observations. First, we found that “opening” of Lck alone is not sufficient to activate TCR signaling but rather that the simultaneous phosphorylation of Tyr^394^ is critical for inducing activation of Lck. In fact, an Lck-biosensor mutant in which the two regulatory tyrosine residues were mutated to phenylalanine (mimicking the primed/non-phosphorylated form of Lck, which represents about 50% of total Lck in primary T cells [2]) is constitutively open and, moreover, that its conformation does not change upon TCR stimulation. Despite its open conformation, this mutant is functionally not active. The second important finding of our study relates to how Lck initiates TCR signaling. Although it is now textbook knowledge that Lck phosphorylates the ITAMs, the molecular mechanisms underlying this phenomenon remain still elusive. In our work, we found that a constitutively open and active Lck mutant (Y505F) does not initiate downstream signaling unless the TCR has been triggered [5]. Therefore, we postulate that TCR stimulation likely induces a conformational change of the CD3 chains allowing the phosphorylation of the ITAMs by Lck (Figure 1). This hypothesis is in line with a proposed model in which antigen-binding stabilizes a conformational change in the TCR/CD3 complex switching the TCR to the so called “active” conformation [6]. How the “active” conformation then promotes phosphorylation of the ITAMs by Lck remains still to be investigated. However, a recently published work proposes that CD3ε can recruit Lck via ionic interactions between the basic residue-rich sequences of CD3ε and acidic residues in the unique domain of Lck [7]. This interaction facilitates the phosphorylation of the ITAMs and hence initiates downstream signaling.

Despite latest advances in our understanding of Lck activation and initiation of TCR signaling, a number of questions still remains open. It would be of particular interest to investigate whether the “active” TCR is involved in the regulation of opening/activation of Lck as suggested by our data. A better understanding of this process is a crucial step towards the development of pharmacological tools to modulate the interplay between the TCR and Lck and, hence, the activation of T cells.
